# The impact of the COVID-19 pandemic on healthy volunteer motivations: a mixed-methods study of participants in plague vaccine trials in the UK and Uganda

**DOI:** 10.1186/s12889-026-27297-1

**Published:** 2026-05-08

**Authors:** Arabella SV Stuart, Richard Muhumuza, Sylvia Kusemererwa, Maria Teddy Ndagire, Matilda Hill, Peter J O’Reilly, Martin Onyango, Wasswa Solomon, Penelope Akankunda, Sylvia Masawi, Shamim Ssendagire, Denis Murphy, Samantha Vanderslott, Christine S Rollier, Eugene Ruzagira, Andrew J Pollard, Nambusi Kyegombe

**Affiliations:** 1 Oxford Vaccine Group, Department of Paediatrics, and the NIHR Oxford Biomedical Research Centre, University of Oxford, Oxford, UK; 2 Uganda Research Unit, Medical Research Council/Uganda Virus Research Institute and London School of Hygiene and Tropical Medicine, Entebbe, Uganda; 3 London School of Hygiene and Tropical Medicine, London, United Kingdom; 4 School of Biosciences, Faculty of Health and Medical Sciences, University of Surrey, Guildford, United Kingdom

**Keywords:** Plague, Vaccine, Motivations, COVID-19, healthy volunteers

## Abstract

**Background:**

The COVID-19 pandemic led to unprecedented interest and participation in vaccine trials globally, and a concurrent increase in vaccine hesitancy. Whether this impacted recruitment of healthy volunteers to subsequent non-COVID vaccine trials is not well studied. We explored the impact of the COVID-19 pandemic on motivations for participating in two clinical trials of the same novel anti-plague vaccine, conducted in the United Kingdom (UK) and Uganda in 2021 and 2022.

**Methods:**

Participants enrolled in PlaVac (UK) and PlaVac Uganda, Phase I trials of ChAdOx1 Plague vaccine, were invited to complete an optional questionnaire and semi-structured interview examining motivations for participating, including questions on the impact of the COVID-19 pandemic on their decision. Questionnaires were self-administered and interviewer-administered for UK and Uganda studies, respectively. Interviews were conducted in local languages, transcribed in English, and analysed using thematic analysis. Results were compared between studies.

**Results:**

Thirty-one of the 45 (68.9%; 25.8% female) UK trial participants and all 36 (100.0%; 27.8% female) of the Uganda trial participants completed questionnaires responses, and 19 Uganda questionnaire respondents completed interviews. Responses to questions on the impact of the COVID-19 pandemic on volunteering decisions were divergent between countries, with little effect for UK participants but a strong positive effect for Ugandan participants. Themes relating to this effect were *“contributor*,* not cause*” in the UK, and in Uganda were *preparedness* (wanting to contribute to vaccine development to prevent suffering and death from future epidemics), *increased awareness* (understanding the vaccine development process and seeing rapidly deployed COVID-19 vaccine trials gave them confidence), and *personal protection* (believing themselves to be protected by the novel plague vaccine). Participants in both studies expressed trust and confidence in the study vaccine which shares the same adenoviral-vectored platform technology used to elicit an immune response (ChAdOx1) with the COVID-19 vaccine ChAdOx1 nCoV-19 (Vaxzevria, AstraZeneca).

**Conclusions:**

For Ugandan participants, COVID-19 and mass vaccination increased knowledge about vaccines and trials and encouraged them to participate in research, but had little impact on UK volunteers. There was no evidence of a negative effect of perceptions of the related ChAdOx1 nCoV-19 vaccine on trial participants’ confidence in the novel plague vaccine’s safety.

**Trial registrations:**

Current controlled trial: ISRCTN41077863, prospective registration date: 19/03/2021, and current controlled trial: ISRCTN79243381, prospective registration date 05/08/2022.

**Supplementary Information:**

The online version contains supplementary material available at 10.1186/s12889-026-27297-1.

## Background

The COVID-19 pandemic of 2020–2023 was a global health crisis unprecedented in modern times, with seven million direct deaths, an excess mortality rate of two to four times this, and associated profound economic and social disruption [1–6]. This prompted a global race to develop vaccines, with over 288 trials registered between 2020 and 2021 [7]. These trials took place in the context of what the World Health Organisation (WHO) described as an “infodemic” of information and misinformation, in addition to countries’ own awareness and health campaigns, and whilst at the time there was a surge in interest vaccine trial volunteering, there was also a rise in vaccine hesitancy, which has persisted beyond the pandemic [8–13]. Early-phase vaccine trials rely on recruiting “healthy volunteers”; people who usually do not stand to benefit from the study intervention, and run the risk of incurring harm from study procedures or adverse reactions [14, 15]. Healthy volunteers’ motivations to participate in vaccine trials are less well studied than those for non-vaccine drug trials, but in diverse income settings have been found to encompass altruistic motivations, desires to contribute to science and financial and non-financial benefits of participation [16–18]. Evidence from studies of COVID-19 trial volunteers in both high- and low-income countries suggests differences to pre-pandemic volunteers, with motivations directly linked to contributing to a mitigation of the social and individual suffering caused by the pandemic, and a sense of urgency for the need to take action in the face of a pressing health crisis [10, 19–21]. Participant motivation is an important aspect of trial participation as it affects recruitment, informed consent, and retention [18, 22–26]. For example, high financial compensation can over-incentivise participation leading volunteers to enrol in trials without due consideration of potential risks, potentially invalidating the concept of informed consent [27, 28]. Non-financial compensation such as free healthcare during study participation has been noted to be particularly incentivising in low-resource settings [29–32]. What is not yet known is whether the COVID-19 pandemic influenced the decision to volunteer for participation in non-COVID-19 vaccine trials, many of which were delayed or suspended during the acute phase of the pandemic in 2020–2021 [33]. 

Whilst COVID-19 is the most recent pandemic of modern times, plague, a lethal disease caused by the bacterium *Yersinia pestis*, tends to be considered a historical disease [34]. This is unfortunately a misconception, as plague is globally distributed in endemic foci, and is classified as a “re-emerging” disease with up to 700 cases annually [35, 36]. Despite this, there is no licenced vaccine approved by the WHO [34, 37]. There is only one plague vaccine still in manufacture, the live-attenuated Russian-produced EV76 NIEEG, and it is used only in countries of the Former Soviet Union due to concerns about its safety, efficacy and reactogenicity [38]. Over 90% of annual cases occur in rural and medically underserved communities in sub-Saharan Africa, including Uganda which contains both endemic foci in Northern regions and is vulnerable to cross-border transmission from the Democratic Republic of Congo [39, 40]. Efforts to develop a modern vaccine have thus far failed [41]. A novel plague vaccine, ChAdOx1 Plague, has been developed by the University of Oxford and underwent Phase I testing in two trials, the first (PlaVac) in Oxford, United Kingdom (UK) starting in late 2021, and the second (PlaVac Uganda) in Masaka, Uganda in early 2022. These trials were positioned in a unique context; both studies began enrolling participants after the commencement of mass COVID-19 vaccination campaigns and the easing of non-pharmaceutical interventions (e.g. social distancing) in their respective countries, and the trial vaccine is closely related to the ChAdOx1 nCoV-19 vaccine (Vaxzevria, AstraZeneca) which had been extensively used in COVID-19 vaccination campaigns in both countries [42–46]. Additionally, recruitment for both studies took place after there had been pauses in multiple countries’ use of the ChAdOx1 nCoV-19 vaccine due to the occurrence of fatal blood clotting events; what later became known as vaccine-induced immune thrombocytopenia and thrombosis (VITT), which garnered extensive media coverage, including in Uganda – and at the time it was unknown what impact this would have on recruitment [47–52]. As part of the PlaVac trials, mixed-methods study procedures comprising a questionnaire and semi-structured interview examining the volunteering motivations and experiences of trial participants were conducted. Data showing the impact of the COVID-19 pandemic on participants motivations and trial experience will be presented here.

## Methods

### Study setting and population

Mixed-methods study procedures were included in two phase I studies of an investigational plague vaccine, ChAdOx1 Plague. PlaVac (ISRCTN:41077863) was a Phase Ia trial (first-in-human, safety and tolerability trial) conducted at the Centre for Clinical Vaccinology and Tropical Medicine, Oxford, UK, and PlaVac Uganda (ISRCTN:79243381) a Phase Ib trial (further assessment of safety and tolerability) conducted by Medical Research Council/Uganda Virus Research Institute and London School of Hygiene and Tropical Medicine Uganda Research Unit field station in Masaka, Southwestern Uganda, where plague is not present. The primary endpoint of both Phase I trials was the safety and tolerability of ChAdOx1 Plague in healthy adults. To be eligible for participation in the PlaVac trials, volunteers had to provide written informed consent, be considered medically healthy, not pregnant or lactating, and adherent to contraception for the duration of the trial. Age inclusion criteria were 18–55 years for the UK study and 18–49 years for the Uganda study. The lower inclusion age-range for the Uganda study was based on the progress of the Uganda COVID-19 vaccination rollout at the time of study design, with the decision to exclude the most prioritised over 50 age group, due to the risk of discencentivising take up of COVID-19 vaccination through study participation as there were temporal exclusions around receiving other vaccines once enrolled in the study.

### Study design, sampling, and sample size

For mixed-methods procedures a convergent parallel design was used. Quantitative and qualitative data were collected concurrently, analysed independently and combined in interpretation [53]. Participation in mixed-methods procedures was optional in both studies, and both studies used the same questionnaire template and topic guide for the semi-structured interviews (with modification of some items for cultural context and language in Uganda versions). For the PlaVac UK study, all participants were invited to complete the questionnaire, with the optional additional completion of an interview, with consecutive sampling. For PlaVac Uganda, all participants were invited to complete the questionnaire, and optionally the interview, but due to budgetary constraints the number of interviews was capped at 20. Of PlaVac Uganda participants who expressed an interest in completing an interview, 20 were purposively sampled for gender, in consecutive order, aiming to include equal numbers of males and females. All participants were reimbursed for study visits, to cover transport and time expenses. For both studies, reimbursement is as per clinical trials unit policy and approved by the local research ethics committee(s) approving the study; reimbursement amounts are estimated to cover local transport costs and time given for study visits. Participants had received information on plague via prior to joining the study via participant information sheets, the informed consent process, and from information-giving sessions run by the trials unit community engagement team in Uganda, and from the study website in the UK.

### Data collection and analysis

For the quantitative component, a 47-item questionnaire was developed based on the literature review, and experience of conducting a survey of COVID-19 trial volunteers (Supplementary Tables 1 and Supplementary Table 2) [10]. Questionnaire domains included demographics, previous experience of clinical trials, attitudes to blood and organ donation, motivations for volunteering for the study, views on risks of the study and experiences of study participation. The questionnaire was reviewed by the PlaVac Uganda study team, with modification of questions for cultural appropriateness. Both questionnaire versions were piloted with local teams before finalisation.

Questionnaire data were collected directly in JISC Online Surveys V2. UK participants completed questionnaires in privacy via a single-use URL sent by the study team. UK responses were pseudo-anonymised through an embedded token in the email link, meaning data was extractable from the survey platform in a fully anonymised format, but participant responses could be tracked. Uganda questionnaires were administered digitally by a researcher who was not previously known to the participant or part of the clinical team, with direct data entry to the online survey using a laptop. There was no linkage of identifiers between mixed-methods procedures and the main study data. Data were analysed descriptively using StataSE V18.0 (StataCorp, Texas, USA) and Microsoft Excel (v2404).

For the qualitative data, an interview guide was developed first for the UK study, based on questionnaire domains and using pre-scripted open-ended questions and prompts, and was reviewed and modified by the Uganda study team for use in PlaVac Uganda (Supplementary Table 3, Supplementary Table 4). For the UK study, potentially sensitive demographics of religion and educational attainment were not collected in the interviews. The guide was piloted among staff at both study sites who were not familiar with the study, to ensure flow and comprehensibility of the prompts. UK interviews were conducted via video call (Microsoft Teams) by a researcher not previously known to the participant. UK interview audio recordings were initially transcribed using the “transcribe” function of Microsoft 365 Word for Web, with generated transcripts listened back to and manually corrected by a researcher. Uganda interviews were conducted in person after questionnaire administration, by the same researcher who conducted the questionnaire. Interviews were conducted in Luganda transcribed verbatim in English and organised using Nvivo 12 Pro (QSR International). Transcripts were fully coded, with codes generated using inductive and deductive approaches. For the Uganda data, transcripts were coded by two researchers, with iterative development of the codebook, and UK transcripts were coded by a single researcher. Codes were then discussed and reviewed between UK and Uganda researchers, with theme generation performed for UK and Uganda data separately, with later comparison between generated themes [54]. 

## Results

### Participants

Enrolment to PlaVac UK took place between August and October 2021, with 45 participants, and enrolment to PlaVac Uganda in March 2022, with 36 participants. Thirty-one of the 45 (68.9%), PlaVac UK participants completed the optional questionnaire and nine of those the interview. All 36 (100%) PlaVac Uganda participants completed the optional questionnaire, all expressed an interest in completing an interview, 20 were purposively sampled from those expressing an interest, and 19 completed an interview (one participant did not attend).

### Quantitative

#### Demographics of questionnaire participants

There was a higher proportion of questionnaire participants in younger age categories in Uganda than in the UK, with 72.2% vs. 48.4% falling in 18–34 years. Both samples were > 70.0% male, which was reflective of the overall study composition (Supplementary Table 5). Uganda participants were predominantly of Christian religions (86.1%), whereas most UK participants had no religion (64.5%). Over half of the Uganda cohort had received only primary education (52.8%), whereas 64.5% of the UK cohort had a university degree. Most participants in both studies were employed (for Uganda participants, employed was defined as having done work in the past 7 days), 94.4% vs. 83.9% in Uganda and UK, respectively. UK participants were more likely to be experienced trial volunteers, with 54.8% (*n* = 17) having previously been in a clinical trial, versus 5.6% (*n* = 2) of the Uganda participants. All UK participants had completed at least eight of the 12 months of study follow-up, and Uganda participants had completed at least five of the seven months (Table 1).

**Table 1:** Demographics of PlaVac UK and Uganda questionnaire participants

	UK (*N* = 31)	Uganda (*N* = 36)
	*n* (%)	*n* (%)
Age category		
18–24 years	8 (25.8%)	13 (36.1%)
25–34 years	7 (22.6%)	13 (36.1%)
35–44 years	6 (19.4%)	9 (25.0%)
45–49/55* years	10 (32.3%)	1 (2.8%)
Gender		
Male	22 (71.0%)	26 (72.2%)
Female	8 (25.8%)	10 (27.8%)
Other	1 (3.2%)	(-)
Religion		
No religion	20 (64.5%)	0 (0.0%)
Christian~	10 (32.3%)	31 (86.1%)
Muslim	0 (0.0%)	5 (13.9%)
Other religion^#^	1 (3.2%)	0 (0.0%)
Ethnicity		
White - British	27 (87.1%)	(-)
White - Other	1 (3.2%)	(-)
Mixed - White and Black Caribbean	1 (3.2%)	(-)
Mixed - Other	1 (3.2%)	(-)
Prefer not to say	1 (3.2%)	(-)
Black African	0 (0.0%)	36 (100.0%)
Education^		
Primary	**(-)**	19 (52.8%)
Secondary school/ up to 16 years (UK)	1 (3.2%)	13 (36.1%)
Higher secondary/further education/more than secondary^$^	10 (32.3%)	4 (11.1%)
University bachelor’s degree	11 (35.5%)	0 (0.0%)
Postgraduate degree	9 (29.0%)	0 (0.0%)
Employment status		
Employed full time	20 (64.5%)	34 (94.4%)
Employed part time	3 (9.7%)	0 (0.0%)
Unemployed and currently looking for work	1 (3.2%)	2 (5.5%)
Student	3 (9.7%)	0 (0.0%)
Homemaker	1 (3.2%)	0 (0.0%)
Self-employed	3 (9.7%)	0 (0.0%)
Type of employment		
Education, law, social, government services	6 (19.4%)	2 (5.6%)
Health	5 (16.1%)	1 (2.8%)
Business/Finance	3 (9.7%)	6 (16.7%)
Carer for other household member	1 (3.2%)	1 (2.8%)
Sales and services	3 (9.7%)	6 (16.7%)
Management	3 (9.7%)	(-)
Natural/applied science	3 (9.7%)	(-)
Student	3 (9.7%)	(-)
Other	4 (12.9%)	(-)
Trades and transport	(-)	12 (33.3%)
Craft and related trades	(-)	3 (8.3%)
Agriculture	(-)	4 (11.1%)
Marital status		
Single (never married)	13 (41.9%)	17 (47.2%)
Married	7 (22.6%)	14 (38.9%)
Separated	0 (0.0%)	3 (8.3%)
Cohabiting	7 (22.6%)	(-)
Divorced	3 (9.7%)	1 (2.8%)
Prefer not to say	1 (3.2%)	0 (0.0%)
Widowed	0 (0.0%)	1 (2.8%)
Number of children		
None	18 (58.1%)	10 (27.8%)
One	5 (16.1%)	7 (19.4%)
Two	6 (19.4%)	4 (11.1%)
Three	2 (6.5%)	2 (5.6%)
Four	0 (0.0%)	6 (16.7%)
Five	0 (0.0%)	2 (5.6%)
Six	0 (0.0%)	2 (5.6%)
Seven	0 (0.0%)	1 (2.8%)
Eight	0 (0.0%)	1 (2.8%)
Prefer not to say	0 (0.0%)	1 (2.8%)
Household income		
Less than £15,000	2 (6.5%)	(-)
£15,000 - £24,999	3 (9.7%)	(-)
£25,000 - £54,999	6 (19.4%)	(-)
£55,000 - £99,999	11 (35.5%)	(-)
£100,000 - £149,999	5 (16.1%)	(-)
£150,000 or more	2 (6.5%)	(-)
Prefer not to say	2 (6.5%)	(-)
Have you ever been in another study before this one?		
Yes	17 (54.8%)	2 (5.6%)
No	14 (45.2%)	34 (94.4%)

* Upper inclusion age range UK/Uganda

~includes Catholic, Protestant and all other Christian denominations

#“Agnostic” free-text

^Education includes completion of any grade within that category

$Includes vocational and professional training

#### Impact of COVID-19 pandemic on volunteering motivations

**Fig. 1:**
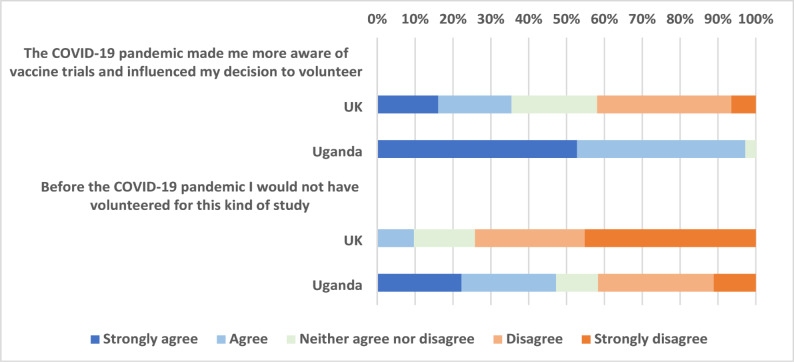
Responses to Likert-type questions on motivations for volunteering for PlaVac studies. COVID-19 related motivations. UK (*n* = 31), Uganda (*n* = 36)

Participants in both studies were asked about the impact of the COVID-19 pandemic on their decision to volunteer for the PlaVac trials (Fig. 1). When asked whether they agreed with the statement *“The COVID-19 pandemic made me more aware of vaccine trials and influenced my decision to volunteer”* only 16.1% (*n* = 5) and 19.4% (*n* = 6) strongly agreed and agreed, respectively (Fig. 1). For the Uganda participants, this was 52.8% (*n* = 19) and 44.4%(*n* = 16), for strongly agree and agree, respectively. UK participants also generally disagreed with the statement *“Before the COVID-19 pandemic I would not have volunteered for this kind of study”* with29.0% (*n* = 9) disagreeing and 45.2% (*n* = 14) strongly disagreeing, whereas Ugandan participants gave mixed responses with almost half in total agreeing (22.2% *n* = 8, “strongly agree”; 25.0% *n* = 9 “agree”), 11.1% (*n* = 4) remaining neutral, and the remainder either disagreeing (30.6%, *n* = 11) or strongly disagreeing (11.1%, *n* = 4).

#### Risks and safety

At the time of enrolment, all PlaVac UK participants had received at least one dose of COVID-19 vaccine, versus 30.6% of PlaVac Uganda participants. Over half of the UK participants had received ChAdOx1-nCoV-19, as had 62.5% of Uganda participants who had received a COVID-19 vaccination (Supplementary Table 5). The vaccination status of the questionnaire and interview participants was not specifically recorded in the mixed-methods dataset (it was collected in the main trial dataset), but some participants discussed their COVID-19 vaccination status at interview.

**Fig. 2:**
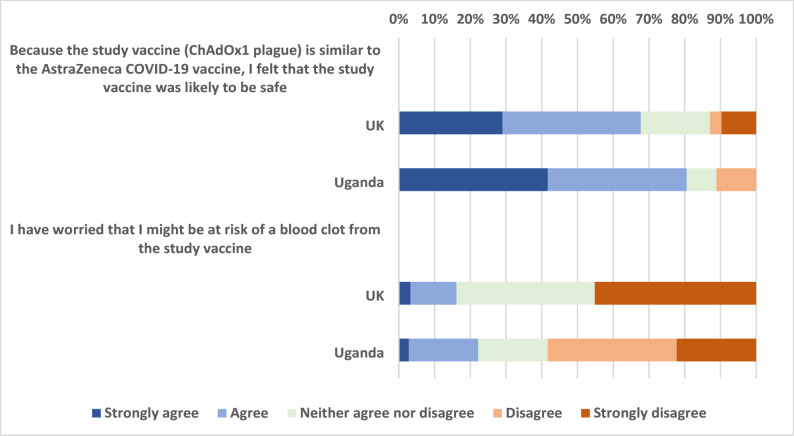
Responses to Likert-type questions on ChAdOx1 plague and relationship to ChAdOx1 nCoV-19 (Vaxzevria, AstraZeneca) vaccine. UK (*n* = 31), Uganda (*n* = 36)

Participants were asked about their perception of the safety of the plague study vaccine in relation to its similarity to the ChAdOx1-nCoV-19 (Vaxzevria, AstraZeneca) COVID-19 vaccine (Fig. 2). Most UK (29.0%, n = 9 strongly agree; 38.7%, n = 12 agree) and Ugandan participants (41.7%, n = 15, strongly agree; 38.9%, n = 14 agree) responded positively to the statement *“Because the study vaccine (ChAdOx1 plague) is similar to the AstraZeneca COVID-19 vaccine*,* I felt that the study vaccine was likely to be safe”.* When asked whether they worried about the risk of vaccine-induced thrombosis with thrombocytopenia (VITT, described to participants as blood clots related to the vaccine) in both studies relatively small proportions agreed they had concerns, at 16.1% (*n* = 5) of UK participants and 22.2% (*n* = 8) of Ugandan participants.

### Qualitative

#### Demographics of interview participants

Due to purposive sampling, the Uganda participants had more even gender balance, whereas UK remained with a male preponderance (66.7%). Older participants were over-represented in UK interviewees, and Uganda interviewees’ ages were more similar to the questionnaire cohort, with the largest age group being 25–34 years (Table 2).

**Table 2:** Demographics of PlaVac UK and Uganda interview participants

	UK (*n* = 9)	Uganda (*n* = 19)
Gender	*n (%)*	*n (%)*
Male	6 (66.7%)	10 (52.6%)
Female	2 (22.2%)	9 (47.4%)
Other	1 (11.1%)	(-)
Age category		
18–24 years	2 (22.2%)	5 (26.3%)
25–34 years	0 (0.0%)	9 (47.3%)
35–44 years	2 (22.2%)	5 (26.3%)
45–55/49* years	5 (55.6%)	0 (0.0%)
Religion		
Christian	-	13 (68.4%)
Muslim	-	5 (26.3%)
Not given	-	1 (5.3%)
Education^	-	
Primary	-	11 (57.8%)
Secondary	-	4 (21.1%)
More than secondary^^	-	4 (21.1%)

- Category not collected

*Upper limit 55years for UK and 49 for Uganda at time of enrolment

^Education includes completion of any grade within that category

^^Includes vocational and professional training

### Themes

Interview themes can be grouped broadly under those relating to impact of the pandemic on motivations, and those relating to risks and safety of trial participation. For impacts on motivations, emergent themes were distinct between studies, whereas with risks and safety there were shared themes between UK and Uganda studies.

### Impacts of the COVID-19 pandemic on volunteering motivations

#### Contributor, not cause (UK)

The COVID-19 pandemic did not emerge as a strong motivator amongst UK participants. When probed by the interviewer, it was described as either having no impact at all, or as a contributing, but not definitive motivator:

*“I don’t know if I would have taken part before [the COVID-19 pandemic]*,* or not taken part*,* I think*,* but I think this* [the pandemic] *definitely pushed me towards a yes*,* I should take part in a vaccine study”.* (UK05, 45-55years)

A single participant described how it has increased his interest in the process of vaccine development:

*“through the pandemic*,* it was like a chance where I wanted to try and help out*,* so I signed up to the St. John’s [St.John’s Ambulance Service – voluntary healthcare organisation assisting with vaccinations]to become a vaccinator*,* to try and expose myself to more of like the clinical side. And I was interested in how vaccines come from being discovered*,* to going through trials*,* to being manufactured*,* and then delivered. I thought the best way to learn was if I could put myself in the different steps and see it from the inside*,* so it sort of played a part*,* yes.”* (UK08, 18-24years, M).

#### Preparedness; “COVID-19 killed many people” (Uganda)

Uganda participants drew a parallel between the abrupt onset and high mortality of the COVID-19 pandemic and the potential for another disease without a ready vaccine, such as plague, to do the same.

*“COVID-19 killed so many people and it also motivated me to participate in the plague vaccine trial to get medication that can help people if there is an outbreak … If there is an outbreak of plague without medication*,* it can also kill so many people as COVID-19 did. ”* (UG34, 25years, M).

Some participants drew a specific link between the risk of being unprepared for an epidemic, and the continued presence of plague in Uganda:

*“Interviewer: do think Plague is a big problem in Uganda or it is not?*

*Interviewee: It is not a big problem because of the low number affected by it but if it gets worse it can be trouble in case we have no vaccine. That is why a vaccine is needed so that we do not get other things like COVID which got us not prepared. Because all is possible” (UG5*,* 26years*,* F)*.

Another element of preparedness was the need to have a vaccine to avoid the use of non-pharmaceutical disease control interventions that caused so much economic and social hardship:

*“It motivated me greatly because the situation was not easy and the way the disease [COVID-19] broke out in a way we did not understand…first we were locked down and not free to move from our places to go to other places. The finances were greatly affected and the disease also claimed a lot of lives every day. Item prices increased rapidly. When someone coughed it would worry you and cause you to distance from them. In case you saw a stranger in the village you would worry about him*,* you would wonder what they are looking for*,* we did not want anyone coming to our home”* (UG29, 35years, F).

#### Increased awareness – “It opened people’s eyes” (Uganda)

Many Ugandan interviewees reported the COVID-19 pandemic had increased their knowledge of the process of vaccine development and clinical trials, and this had positively influenced their decision to volunteer. Interviewees reported being sensitised through COVID-19 vaccination information campaigns on television and radio, which had increased their confidence, enabling them to act on their desire to contribute to the protection of their communities:

*“COVID scared me and I refused to get vaccinated. It was during COVID that I heard on the radio about the process of first trying the vaccine on animals and later on people. I heard that on the radio before coming here for the study. When the study came into my community it helped me to understand more and I decided to participate because it helped me to learn to contribute. After all*,* you or your family could get that disease or anyone in the community. Like the way COVID broke out in the community. So*,* I decided to participate so that researchers can do their best as we contribute through participation*” (UG25, 36years, F).

This increased knowledge and awareness also affected participants’ perception of the safety of vaccine trials, both through being told about the development process, and through witnessing others be safely vaccinated:

“Yes, it opened people’s eyes to understand that trying vaccines on you does not kill you. As they used to think about it. Because even the COVID-19 vaccine had been tried on people in a short time” (UG11, 26years,F)

“Interviewer: Do you think COVID increased awareness about vaccine trials?

Interviewee: Yes sir, It greatly contributed to vaccine trial awareness because I expect that all that accepted to participate in this study greatly relied on that. And the time they sent us the COVID vaccine, you would know that there are also people who stood in for us or who surrendered themselves to get the vaccine. It was equally tried on people. They just did not get it out of the blue and injected it in people. And we did not get news that when that vaccine was tried on a group of people in a particular area they were harmed. We did not get that kind of news” (UG29, 35years, F)

#### Personal protection – “I will be safe” (Uganda)

Participants are explicitly counselled during the consent process that the vaccine is new, and it is unknown whether it would offer any protection against plague disease. However, in both interviews and free-text responses to the questionnaire, some participants (both young and old) were motivated to participate because they believed receiving the novel plague vaccine would protect them from a future outbreak. This need for personal protection was also explicitly linked to their experiences of the COVID-19 pandemic:

*“My major reason was COVID-19 because I heard it affected many people and many died so I decided to also get vaccinated because in case of an outbreak. We were told the plague had broken out before. I went and asked my elder sibling who also told me the plague was here in the early days. I thought if I take this opportunity to get vaccinated even when it breaks out*,* I will be safe”(*UG25,36y, F).

*“To see that I help others and also help myself by getting a vaccine to prevent myself from getting plague … The reason for the way I saw people dying and the condition in which they were living. Indeed*,* and when a chance came and there was a trial*,* I said let me give in myself to try this in that even when this breaks out. I can survive it”* (UG36, 29years, M).

Whilst most participants describing “personal protection” felt it was likely the vaccine would be effective, a few were aware that it could fail, but still felt it was worth having:

*“I was greatly motivated by the fact that back in the day we had been hit by COVID. The situation we went through was not good. Now when they taught us about participating in studies*,* it’s the truth that someone has got to fight for themselves and also fight for his nation…If the vaccine gets to be successful. It will have saved me and also save others.’’* (UG29, 35y, F).

*“The other thing is that I expected that if I come and God helps me and this vaccine succeeds it means that even when Plague breaks out. I will not be worried because I will be aware that I was vaccinated. If this vaccine works my body will be able to have the antibodies that fight plague”* (UG22, 23y, M).

#### Risks and safety

Interview responses from both studies elucidated reasonings behind responses on the link between the two ChAdOx1 vaccines and the perceived risk of the trial, with themes of “safer because ChAdOx1” and “negative press and rumours” emerging.

#### Safer because ChAdOx1 – “it’s the same thing” (UK & Uganda)

Many interviewees in UK and Uganda studies drew a link between the use of ChAdOx1-nCoV-19 as a pandemic vaccine, and the potential riskiness of the plague trial vaccine; with most participants feeling that this made it safer. In keeping with the difference in proportions previously vaccinated with ChAdOx1 nCoV-19 between the two studies, UK interviewees were more likely to link the experience of being personally ChAdOx1 nCoV-19 vaccinated with the perception of safety, whereas Uganda participants were more likely to report witnessing its use in their communities as the reassuring factor; although some interviewees had been also vaccinated with ChAdOx1 nCoV-19 and found this personally reassuring .

*“I had previously had a ChAdOx1 vaccine against COVID*,* so obviously*,* so there’s one thing there which is sort of like*,* you know*,* I have been injected with this vector before. So that’s a few of the things that you might imagine that might go wrong*,* that you could*,* that even just for me*,* I have had experience of having that vaccination so*,* you know*,* I’m thinking I look*,* I was looking at it*,* as this is a sort of*,* this is basically a fairly similar vaccination with a slightly different bit of… A slightly different bit pasted in. So I was assuming the risk was fairly low”* (UK06, 45-55years, M).

*“It made me stronger in a way that if the vaccine is in any way related to that of COVID. Those vaccinated against COVID are many and I did not hear that someone died because of getting vaccinated” (UG29*,* 35y*,* F)*.

#### Negative press and rumours – “these things did not happen” (UK & Uganda)

The impact of negative press and rumours surrounding COVID-19 vaccination was discussed by participants in relation to the study vaccine. Only a single participant in the UK study, mentioned hearing about negative events related to the ChAdOx1-nCoV-19 vaccine in the media, but this did not affect their confidence that the vaccine platform would be safe:

*“Like I don’t believe I’ll be an anomaly* [have a rare side effect]. *I don’t think I’ll have adverse reactions to it. So I didn’t*,* I don’t think I ever really thought that it was a bad thing that it was based off of that*,* due to that history* [of episodes of VITT reported in media]. *I thought it was more of a good thing because it’s based off of that* [ChAdOx1 vaccine platform], *and that that’s something that’s been working*,* you know? … So I don’t think it made me think any worse of it just because of what media has said because you can’t trust everything in the media”* (UK03, 18-24years, F).

Participants in the Uganda study did report rumours they had heard about COVID-19 vaccination, and many had been personally afraid of being vaccinated:

*“We were scared of death. We were suspicious of the vaccine.”* (UG35,40years, M).

The most common rumours were that the vaccine would kill you, or would cause sterility and was a method of population control:

*“I thought that the vaccine would kill me. Okay at first we were so fearful. You could think that what if they inject me and the vaccine is aimed at reducing on the number of people in the country”* (UG01,26years, M).

Key in alleviating fears about COVID-19 vaccination was witnessing others not experience adverse effects from vaccination, and this gave confidence to participate in the PlaVac trial:

*“…for COVID-19 I was even scared of the injection [vaccine] as people said the moment you get the injection you are going to die. My children went first for that vaccine but when they came back home I saw that nothing had happened to them so I decided to also go. That is why even when this trial came I wasn’t scared because I said even with COVID nothing happened when they tried the vaccine on us”* (UG24, 31years, F).

For some, seeing that rumours they heard about COVID-19 vaccines were not true in combination with increased knowledge of the process of vaccine development alleviated concerns about joining the study:

*“There is a way it helped me because before there were rumours that COVID-19 vaccines would kill people and that is not what happened. He mentioned more negative effects that would result from getting vaccinated with COVID and they were terrible*,* but when we got vaccinated*,* those things did not happen. Yet the vaccine was made very fast and not studied as those at MRC. In my thinking I said the vaccines tried through MRC are given time and even tried through animals and later people to make sure that they are safer”* (UG5, 26years, F).

One Ugandan participant did express concern regarding the plague vaccine sharing the same platform as the COVID-19 vaccine, worrying they could be vaccinated by stealth through the trial:

*“I just heard it when they talked about it*,* but I was not convinced because I was not vaccinated … It worried me because I said they could be wanting to vaccinate us through PlaVac. The way we had refused to be vaccinated”* (UG1,26years, M).

No participants in either study specifically discussed concerns about the risk of experiencing VITT.

## Discussion

This study presents evidence of the impacts of the COVID-19 pandemic on healthy volunteers’ motivations and perceptions of a novel anti-plague vaccine, ChAdOx1 Plague, from early-phase vaccine trials conducted in the UK and Uganda.

The UK and Uganda PlaVac studies took place at different times in relation to the COVID-19 pandemic, with the UK study taking place very shortly after the completion of the UK COVID-19 vaccine rollout in 2021 and with some restrictions still in place, and PlaVac Uganda commencing in February 2022, when COVID-19 cases were falling, restrictions were easing, but vaccination rollout had reached only 10% of the population [42, 46]. There were marked differences between the studies in the effect of the pandemic on motivations for volunteering, with a much clearer positive effect of the COVID-19 pandemic on Ugandan participants’ decisions. Fewer than a third of UK questionnaire participants agreed that the pandemic influenced their decision to volunteer, and most agreed they would have participated in this kind of study pre-pandemic, with responses similar amongst the UK interviewees, who mainly described the COVID-19 pandemic as contributory to their decision, but not pivotal. This contrasts with the almost universal agreement amongst Uganda questionnaire participants that the COVID-19 pandemic had increased their awareness of vaccine trials and 41.7% reporting they would not have volunteered before it. It is unclear what the precise difference in timing may have had on people’s motivation to join the studies between the two countries, however it has been clearly documented that there was a surge in interest in volunteering for COVID-19 vaccine trials during the pandemic [55, 56]. This may have had a “knock-on” effect on participants volunteering for PlaVac Uganda due to exposure to education and awareness campaigns in relation to COVID-19 vaccination. The “Ekya COVID-19 Kijja Kugwa” (“COVID-19 Will Pass”) was a national multimedia campaign launched in late 2021 in Uganda and included information on vaccine safety and addressing vaccine hesitancy [13]. Many interviewees reported that learning about the process of vaccine development through COVID-19 campaigns had altered their perception of vaccine trials and eased fears about participating in research, the theme of “increased awareness”.

It is also possible that the difference in the effect of the COVID-19 pandemic on motivations between the studies is related to the large difference in prior trial participation. Whilst over half of UK participants had been in another trial before, for the Uganda participants, this was only 5.6%. Evidence from high-income settings is that serial trial participation is common [27, 57–59]. Amongst volunteers for Phase I drug studies in the United States (US), serial trial participation is strongly influenced by study financial compensation, with some even using it as a primary source of income, and this has raised concerns around whether habituation and financial incentives can invalidate informed consent or the element of free choice in volunteering [58, 59]. Financial inducement may be less of a factor in influencing repeat participation in the UK participants in this study, as they appear to be from a higher socioeconomic groups than is usually reported for healthy volunteers [60–62]. 

A key question relating to this work is whether the likelihood of oneself or one’s community being affected by an outbreak or epidemic of the disease the study vaccine is targeting has an influence on the decision to volunteer, and there is little evidence exploring this. One study of Canadian healthy volunteers compared the motivations of those who had participated in an influenza vaccine trial versus an Ebola vaccine trial recruiting during the 2014-15 West African Ebola epidemic, and found generally similar “altruistic” motivations in both groups; although media coverage of the Ebola epidemic had had some impact on volunteering for the Ebola study [63]. This question was not directly addressed in the questionnaire, and UK interview participants did not discuss this, but for the Uganda interviewees there was a clear link between experiences of the COVID-19 pandemic and their perceived benefits of potentially preventing similar future disease outbreaks in their communities (“preparedness”) through participating in vaccine research, and also in some cases through receiving personal benefit from the investigational vaccine (“I will be safe”). This may have been influenced by study information and consent materials which stated that plague is still present in Uganda (in a different region to the study site). Uganda participants’ experiences of living through the COVID-19 pandemic had left a marked impression of the vulnerability of society to unexpected epidemics; and not just the direct consequences of illness and death, but the social and economic disruption which came with non-pharmaceutical efforts to control COVID-19 in Uganda [64–66]. To counter this, participants felt that contributing to vaccine research by becoming a trial participant was an important way to protect themselves, their families, communities and nation from future harm (“preparedness”). This sentiment echoes the views of volunteers for COVID-19 vaccine trials, who were strongly motivated by a wish to alleviate the suffering and harms being caused by the pandemic which they were living through; but is different in that PlaVac participants had no lived-experience of plague [10, 20, 67, 68]. 

A unique factor of the PlaVac trials was that they were utilising the ChAdOx1 adenoviral-vector vaccine platform, at the same time that the ChAdOx1 nCoV-19 (Vaxzevria, AstraZeneca) was being used to vaccinate millions of people globally [43]. Despite widespread media coverage of the association of the related ChAdOx1 nCoV-19 with VITT, and being specifically counselled on the potential for VITT to be associated with ChAdOx1 Plague, participants in both UK and Uganda PlaVac studies reported that the investigational plague vaccine being similar to the COVID-19 vaccine increased their confidence in its safety (“safer because ChAdOx1”), and none mentioned concerns about blood clots. This might have been expected for the UK cohort, where over half had already been vaccinated with ChAdOx1 nCoV-19, but only eight of the Uganda cohort had received it and still were highly reassured by the similarity. Over half of UK participants, being repeat trial volunteers, may have also positively affected the perception of the safety of the study vaccine, as evidence suggests that repeated clinical trial volunteering tends to lower perceptions of study riskiness, but this doesn’t explain the lack of concern amongst Uganda participants [69]. It also must be considered that these cohorts were self-selecting, and those with concerns about safety of ChAdOx1 Plague were unlikely to volunteer.

Another interesting finding on the impact of the COVID-19 pandemic was that some Uganda participants reported initially having had concerns about COVID-19 vaccines, and been somewhat vaccine hesitant, but this had changed during vaccination campaigns (“negative press and rumours”). Uganda interviewees described witnessing others be vaccinated without succumbing to any of the conditions that were rumoured to be caused by COVID-19 vaccines, such as death or infertility (common myths circulating in Uganda at the time), increasing their confidence that fears about receiving a study vaccine might also be unfounded [70]. 

### Limitations

There are some important limitations when considering these results. Whilst all efforts were made to modify the data collection tools to be linguistically and culturally appropriate for the Uganda study, the original versions were designed for the UK context, and questions may not have transposed well. Additionally, an important difference was the questionnaires being administered in the Uganda study, which may have increased social-desirability and courtesy bias in the results [71]. Not all study participants completed the mixed-methods procedures, with uptake particularly low in the UK study, which may have biased the results towards the opinions of a group of participants who had more positive experiences of participation. Relatively few UK participants participated in the interviews, and as the Uganda study sample size was pre-specified data saturation was not used to decide when to stop the semi-structured interviews. It is also important to note that the findings presented here on COVID-19 did not stand in isolation from other factors influencing participation such as altruism, trust, impact of study benefits and perceptions of risk, which are beyond the scope of this paper. Whilst these data provide insights as to the impact of the COVID-19 pandemic on the decision of individuals who volunteered for and were enrolled in a vaccine trial, it does not capture what the impact may have been on people who decided not to volunteer, or who withdrew during the screening process. Ultimately these data represent only the views of a small fraction of the general population, the majority of whom do not participate in clinical research.

## Conclusions

Whilst the long-term impact of the pandemic on vaccine trials is as yet unknown, these data provide valuable insights as to the impact of COVID-19 on volunteers participating in studies of the same novel plague vaccine, in both the UK and Uganda. These results suggest that there may be less of a “post-pandemic effect” in the high- than low-income setting in increasing vaccine trial participation. There was a noticeable difference in the effect of the pandemic as a motivator between studies, with both the experience of the COVID-19 pandemic and associated mass vaccination campaigns increasing Ugandan participants’ knowledge about vaccines and trials and encouraging them to participate in research, whereas UK participants were more likely to have participated in clinical research before and to report that the COVID-19 pandemic had not affected their decision. Despite major safety events being linked to the COVID-19 vaccine ChAdOx1 nCoV-19, its relationship to the study vaccine was generally seen as a positive, and increased participants confidence in the safety of the study vaccine, mainly through participants witnessing its use in COVID-19 mass vaccination campaigns.

## Supplementary Information

Supplementary Material 1.

Supplementary Material 2.

## Data Availability

The datasets used and analysed during the current study are available from the corresponding author on reasonable request.

## Abbreviations

ChAdOx1: Chimpanzee adenovirus Oxford 1
COVID: 19–Coronavirus infectious disease 2019
ISRCTN: International Standard Randomised Controlled Trial Number
UK: United Kingdom
US: United States
VITT: Vaccine–induced thrombosis with thrombocytopenia
WHO: World Health Organisation
