# Supplementary material for: The impact of the COVID-19 pandemic on healthy volunteer motivations: a mixed-methods study of participants in plague vaccine trials in the UK and Uganda
Source: BMC Public Health. 2026 May 8;26:2006. doi: 10.1186/s12889-026-27297-1 (PMC13326140; doi:10.1186/s12889-026-27297-1)
Supplement: Supplementary file 1 — Supplementary Material 1. [file 12889_2026_27297_MOESM1_ESM.docx]

# Supplementary appendix

Supplementary Table 1 PlaVac UK questionnaire

| Domain name | Questions | Response options |
| --- | --- | --- |
| Section A - Demographics | |  |
| Instruction text | This part of the survey will ask you for some general background information.  Please note that your responses here are anonymised, and are not directly tied to your main plague study number. | |
|  | What was your age group when you joined the plague study? | 18-24  25-34  35-44  45-55 |
|  | Please select an option below that best describes your ethnicity | White - British  White - Irish  White - Other  Black or Black British - Caribbean  Black or Black British - African  Black or Black British - Other  Asian or Asian British - Indian  Asian or Asian British - Pakistani  Asian or Asian British - Bangladeshi  Asian or Asian British - Chinese  Asian or Asian British - Other Asian background  Mixed - White and Black Caribbean  Mixed - White and Black African  Mixed - White and Asian  Mixed - Other  Gypsy or Traveller  Arab  Other___________  Prefer not to say |
|  | What is your religion? | No religion  Christian (including Church of England, Catholic, Protestant and all other Christian denominations)  Buddhist  Hindu  Jewish  Muslim  Sikh  Other____________  Prefer not to say |
|  | What is your gender? | -Male  -Female  - Other  -Prefer not to say |
|  | What is the highest level of education you have completed? | Primary school  Secondary school up to 16 years (e.g GCSE)  Higher secondary or further education (e.g. A-levels, BTEC, IB)  University bachelor’s degree  Postgraduate degree  Other____________  Prefer not to say |
|  | What is your current employment status? | Employed full time (35 or more hours per week)  Employed part time (up to 39 hours per week)  Unemployed and currently looking for work  Unemployed and not currently looking for work  Student  Retired  Homemaker  Self-employed  Unable to work  Other___________  Prefer not to say |
|  | What type of work do you usually do? | Management  Business/Finance  Natural/applied science  Health  Education, law, social, government services  Sales and services  Trades and transport  Student  Unemployed  Retired  Carer for other household member  Other__________  - Prefer not to say |
|  | When you joined the study, what was your marital status? | Single (never married)  Married  Civil partnership  Cohabiting  Separated  Divorced  Widowed  Other__________  Prefer not to say |
|  | In which of these groups is your yearly household income? | Less than £15,000  £15,000 - £24,999  £25,000 - £54,999  £55,000 - £99,999  £100,000 - £149,999  £150,000 or more  Prefer not to say |
|  | How many children do you have? | None  1  2  3  4  More than 4  Prefer not to say |
|  | Approximately how many months of this study have you completed? | ____________  (enter whole number) |
| Section B – Previous experience of clinical trials/how they heard about the study | |  |
|  | Have you ever been in another study before this one? | Yes  No |
|  | If yes, how many other studies have you participated in? | 1  2  3  4  5+ |
|  | If yes, what was the type of study? | -Studies involved a drug/vaccine  -Studies had no drug/vaccine (e.g. psychological study or imaging study)  -A mixture |
|  | Before this study, what was your knowledge of clinical trials? | I had never heard of them  I knew a little bit about them  I knew quite a bit about them  I knew a lot about them  I work, or have worked, in clinical trials  I work, or have worked, in research |
|  | How did you hear about this study? | Friend  University email  Internet search  OVG website  OVG newsletter  TV  News article  Social media  Other_________ |
|  | If social media, which form? | Facebook  Twitter  Instagram  WhatsApp  Other |
|  | Did you discuss with anyone else about your decision to volunteer for the study? | Yes  No |
|  | (If yes) Who did you discuss it with? (Select all that apply) | Partner/spouse  Children  Other family  Friends  Colleagues |
|  | (If yes) When you discussed volunteering, were the responses you received: | Mostly positive  Neutral  Mostly negative |
| Section C – Organ/blood donation attitudes | |  |
|  | I am currently or have been previously a blood donor | Yes  No |
|  | If I died, I would like my organs to be donated | Yes  No  Maybe |
| Section D – Motivations for volunteering | |  |
| Instruction text | The following questions will ask you about your reasons for volunteering for this study.  Rate each of the statements based on how much you agree with them. | |
|  | I wanted to help others | Strongly agree  Agree  Neither agree nor disagree  Disagree  Strongly disagree |
|  | I wanted to receive the financial reimbursement for participating in the study | Strongly agree  Agree  Neither agree nor disagree  Disagree  Strongly disagree |
|  | I wanted to get a medical check-up to find out more about my own health | Strongly agree  Agree  Neither agree nor disagree  Disagree  Strongly disagree |
|  | I wanted to contribute to medical and scientific progress | Strongly agree  Agree  Neither agree nor disagree  Disagree  Strongly disagree |
|  | I was aware of the public health impact of plague and wanted to make a difference | Strongly agree  Agree  Neither agree nor disagree  Disagree  Strongly disagree |
|  | I was curious about the experience of being in a clinical trial | Strongly agree  Agree  Neither agree nor disagree  Disagree  Strongly disagree |
|  | The COVID-19 pandemic made me more aware of vaccine trials and influenced my decision to volunteer | Strongly agree  Agree  Neither agree nor disagree  Disagree  Strongly disagree |
|  | Before the COVID-19 pandemic I would not have volunteered for this kind of study | Strongly agree  Agree  Neither agree nor disagree  Disagree  Strongly disagree |
|  | I felt that others would view my participation positively | Strongly agree  Agree  Neither agree nor disagree  Disagree  Strongly disagree |
|  | Did you have any other motivations or reasons for volunteering for the study? | Yes  No |
|  | If yes, please describe | Freetext |
|  | Do you think that the type of disease a study vaccine is for would have any effect on how likely you were to volunteer for that study? | Yes  No |
|  | Can you explain why? | Freetext |
| Section E – Views on risks of this study | |  |
|  | When you volunteered for the study, how risky for your own health did you think being in a vaccine trial might be? | 1 – Not at all risky  2 – Slightly risky  3 – Somewhat risky  4 – Moderately risky  5 – Risky  6 – Very risky  7 – Extremely risky |
|  | Did your opinion of the riskiness of the study change after your screening visit and the informed consent process? | Yes  No |
|  | If yes, did you feel the trial was more or less risky after your screening visit? | More risky  Less risky |
|  | *(If yes or no)* Can you explain why? | (Freetext) |
| Instruction text | Please rate the following statements based on how much you agree with them | |
|  | I have worried that I could have a severe side-effect from the study vaccine | Strongly Agree  Agree  Neither agree nor disagree  Disagree  Strongly Disagree |
|  | I have worried that I might be at risk of a blood clot from the study vaccine | Strongly Agree  Agree  Neither agree nor disagree  Disagree  Strongly Disagree |
|  | I have worried that there might be long-term health effects from the study vaccine | Strongly Agree  Agree  Neither agree nor disagree  Disagree  Strongly Disagree |
|  | Because the study vaccine (ChAdOx1 plague) is similar to the AstraZeneca COVID-19 vaccine, I felt that the study vaccine was likely to be safe. | Strongly Agree  Agree  Neither agree nor disagree  Disagree  Strongly Disagree |
|  | Because this study was being run by the University of Oxford, I felt that it would be safe. | Strongly Agree  Agree  Neither agree nor disagree  Disagree  Strongly Disagree |
|  | My experience of the potentially negative aspects of study participation (e.g. inconvenience of visits, pain from blood sampling and vaccines, side effects) was: | Worse than I expected  About the same as what I expected  Better than I expected |
|  | Can you explain why you chose this answer? | Freetext |
|  | The information given at the start of the study about potentially negative aspects of study participation (e.g. inconvenience of visits, pain from blood sampling and vaccines, side effects) was accurate | Strongly Agree  Agree  Neither agree nor disagree  Disagree  Strongly Disagree |
|  | I trusted the skills of the clinical staff working on the study | Strongly Agree  Agree  Neither agree nor disagree  Disagree  Strongly Disagree |
|  | I felt safe during the study | Strongly Agree  Agree  Neither agree nor disagree  Disagree  Strongly Disagree |
|  | I felt confident that if I had a medical problem during the study I would be looked after | Strongly Agree  Agree  Neither agree nor disagree  Disagree  Strongly Disagree |
|  | Based on my experiences, I would encourage others to volunteer for a clinical trial | Strongly Agree  Agree  Neither agree nor disagree  Disagree  Strongly Disagree |
|  | I would have volunteered for this study even if there was no financial compensation | Strongly Agree  Agree  Neither agree nor disagree  Disagree  Strongly Disagree |
|  | The amount of financial compensation for participation in the trial was appropriate. | Strongly Agree  Agree  Neither agree nor disagree  Disagree  Strongly Disagree |
|  | I would consider taking part in another vaccine trial | Strongly Agree  Agree  Neither agree nor disagree  Disagree  Strongly Disagree |
|  | What has been your overall experience of the study? | It has been an entirely positive experience  It has been a mostly positive experience  It has been a neutral experience  It has been a mostly negative experience  It has been an entirely negative experience |
|  | What was the best part of being in the study for you? | Freetext |
|  | What was the worst part of being in the study for you? | Freetext |

Supplementary Table 2 PlaVac Uganda questionnaire

| Domain name | Questions | Response options |
| --- | --- | --- |
| Section A - Demographics | |  |
| Instruction text | This part of the survey will ask you for some general background information. | |
|  | What was your age group when you joined the plague study? | 18-24  25-34  35-44  45-50 |
|  | What is your religion? | No religion  Christian (including Catholic, Protestant and all other Christian denominations)  Muslim  Traditional  Other  Prefer not to say |
|  | What is your gender? | -Male  -Female  -Prefer not to say |
|  | What is the highest level of education you have completed? | No education  Primary  Secondary  More than secondary  Other____________  Prefer not to say |
|  | What is your current employment status?  (“Currently employed” is defined as having done work in the past 7 days. Includes persons who did not work in the past 7 days but who are regularly employed and were absent from work for leave, illness, vacation, or any other such reason.) | Currently employed  Not currently employed  Student  Retired  Homemaker  Unable to work  Other___________  Prefer not to say |
|  | What type of work do you usually do? | Management  Business/Finance  Agriculture  Science  Health  Education, law, social, government services  Sales and services  Trades and transport  Plant and machine operators  Craft and related trades  Student  Unemployed  Retired  Carer for other household member  Other__________  - Prefer not to say |
|  | When you joined the study, what was your marital status? | Single (never married)  Married  Living together  Separated  Divorced  Widowed  Other__________  Prefer not to say |
|  | How many children do you have? | None  1  2  3  4  5  6  7  8  More than 8  Prefer not to say |
|  | Approximately how many months of this study have you completed? | ____________  (enter whole number) |
| Section B – Previous experience of clinical trials/how they heard about the study | |  |
|  | Have you ever been in another study before this one? | Yes  No |
|  | If yes, how many other studies have you participated in? | 1  2  3  4  5+ |
|  | If yes, what was the type of study? | -Studies involved a drug/vaccine  -Studies had no drug/vaccine (e.g. questionnaire study, or study where samples were taken but no intervention given)  -A mixture |
|  | Before this study, what was your knowledge of clinical trials? | I knew nothing about them  I knew a little bit about them  I knew quite a bit about them  I knew a lot about them  I work, or have worked, in clinical trials  I work, or have worked, in research |
|  | How did you hear about this study? | From a friend  Community outreach  Health worker  Study staff  Other_________ |
|  | Did you discuss with anyone else about your decision to volunteer for the study? | Yes  No |
|  | (If yes) Who did you discuss it with? (Select all that apply) | Partner/spouse  Children  Other family  Friends  Colleagues |
|  | (If yes) When you discussed volunteering, were the responses you received: | Mostly positive  Neutral  Mostly negative |
| Section C – Organ/blood donation attitudes* | |  |
|  | I have donated blood before | Yes  No |
|  | I would consider donating blood in future | Yes  No  Maybe |
| Section D – Motivations for volunteering | |  |
| Instruction text | The following questions will ask you about your reasons for volunteering for this study.  Rate each of the statements based on how much you agree with them. | |
|  | I wanted to help others | Strongly agree  Agree  Neither agree nor disagree  Disagree  Strongly disagree |
|  | I wanted to receive the financial reimbursement for participating in the study | Strongly agree  Agree  Neither agree nor disagree  Disagree  Strongly disagree |
|  | I wanted to get a medical check-up to find out more about my own health | Strongly agree  Agree  Neither agree nor disagree  Disagree  Strongly disagree |
|  | I wanted to contribute to medical and scientific progress | Strongly agree  Agree  Neither agree nor disagree  Disagree  Strongly disagree |
|  | I was aware of the public health impact of plague and wanted to make a difference | Strongly agree  Agree  Neither agree nor disagree  Disagree  Strongly disagree |
|  | I was curious about the experience of being in a clinical trial | Strongly agree  Agree  Neither agree nor disagree  Disagree  Strongly disagree |
|  | The COVID-19 pandemic made me more aware of vaccine trials and influenced my decision to volunteer | Strongly agree  Agree  Neither agree nor disagree  Disagree  Strongly disagree |
|  | Before the COVID-19 pandemic I would not have volunteered for this kind of study | Strongly agree  Agree  Neither agree nor disagree  Disagree  Strongly disagree |
|  | I felt that others would view my participation positively | Strongly agree  Agree  Neither agree nor disagree  Disagree  Strongly disagree |
|  | Did you have any other motivations or reasons for volunteering for the study? | Yes  No |
|  | If yes, please describe | Free text |
|  | Do you think that the type of disease a study vaccine is for would have any effect on how likely you were to volunteer for that study? | Yes  No |
|  | Can you explain why? | Free text |
| Section E Knowledge of and attitude to plague** | | |
|  | Before joining this study, had you heard of plague disease? | Yes  No |
|  | (if yes) From where had you heard about plague? | From family  From friends  In school  Through work  From healthcare workers/clinics  From the internet  From the radio  From a traditional healer  From a pharmacy/drug shop |
|  | Before joining this study, did you know there was plague in Uganda? | Yes  No |
|  | Do you know which parts of Uganda plague is usually in? | Northern  Southern  Western  Central  North west  South West  North East  South East |
|  | Do you know which of these things are ways in which you can catch plague? (can select multiple responses) | From a mosquito bite  From drinking dirty water  From a flea bite  From touching a person with plague |
|  | Do you know which of these can be symptoms of plague? | Fever  Painful swellings in the groin and armpits or neck  Weight loss over months  Coughing up blood  Pain with urination |
|  | Do you know if there are any treatments for plague? | Yes  No |
| a. | (If yes) Can you say what the treatment is for plague? | Free text |
|  | Can plague be a fatal disease? | Yes  No  Don’t know |
|  | Do you think if there was an outbreak of plague where you live people would be willing to take a new vaccine for it? | Yes  No  Not sure |
| Section F – Views on risks of this study | |  |
|  | When you volunteered for the study, how risky for your own health did you think being in a vaccine trial might be? | 1 – Not at all risky  2 – Slightly risky  3 – Somewhat risky  4 – Moderately risky  5 – Risky  6 – Very risky  7 – Extremely risky |
|  | Did your opinion of the riskiness of the study change after your screening visit and the informed consent process? | Yes  No |
|  | If yes, did you feel the trial was more or less risky after your screening visit? | More risky  Less risky |
|  | *(If yes or no)* Can you explain why? | (Freetext) |
| Instruction text | Please rate the following statements based on how much you agree with them | |
|  | I have worried that I could have a severe side-effect from the study vaccine | Strongly Agree  Agree  Neither agree nor disagree  Disagree  Strongly Disagree |
|  | I have worried that I might be at risk of a blood clot from the study vaccine | Strongly Agree  Agree  Neither agree nor disagree  Disagree  Strongly Disagree |
|  | I have worried that there might be long-term health effects from the study vaccine | Strongly Agree  Agree  Neither agree nor disagree  Disagree  Strongly Disagree |
|  | Because the study vaccine (ChAdOx1 plague) is similar to the AstraZeneca COVID-19 vaccine, I felt that the study vaccine was likely to be safe. | Strongly Agree  Agree  Neither agree nor disagree  Disagree  Strongly Disagree |
|  | Because this study was being run by the MRC/UVRI, I felt that it would be safe. | Strongly Agree  Agree  Neither agree nor disagree  Disagree  Strongly Disagree |
|  | My experience of the potentially negative aspects of study participation (e.g. inconvenience of visits, pain from blood sampling and vaccines, side effects) was: | Worse than I expected  About the same as what I expected  Better than I expected |
|  | Can you explain why you chose this answer? | Freetext |
|  | The information given at the start of the study about potentially negative aspects of study participation (e.g. inconvenience of visits, pain from blood sampling and vaccines, side effects) was accurate | Strongly Agree  Agree  Neither agree nor disagree  Disagree  Strongly Disagree |
|  | I trusted the skills of the medical staff working on the study | Strongly Agree  Agree  Neither agree nor disagree  Disagree  Strongly Disagree |
|  | I felt safe during the study | Strongly Agree  Agree  Neither agree nor disagree  Disagree  Strongly Disagree |
|  | I felt confident that if I had a medical problem during the study I would be looked after | Strongly Agree  Agree  Neither agree nor disagree  Disagree  Strongly Disagree |
|  | Based on my experiences, I would encourage others to volunteer for a clinical trial | Strongly Agree  Agree  Neither agree nor disagree  Disagree  Strongly Disagree |
|  | I would have volunteered for this study even if there was no financial compensation | Strongly Agree  Agree  Neither agree nor disagree  Disagree  Strongly Disagree |
|  | The amount of financial compensation for participation in the trial was appropriate. | Strongly Agree  Agree  Neither agree nor disagree  Disagree  Strongly Disagree |
|  | I would consider taking part in another vaccine trial | Strongly Agree  Agree  Neither agree nor disagree  Disagree  Strongly Disagree |
|  | What has been your overall experience of the study? | It has been an entirely positive experience  It has been a mostly positive experience  It has been a neutral experience  It has been a mostly negative experience  It has been an entirely negative experience |
|  | What was the best part of being in the study for you? | Freetext |
|  | What was the worst part of being in the study for you? | Freetext |
| * Question removed from this section **“**If I died, I would like my organs to be donated” – considered to be culturally sensitive.  ** Section added, not present on UK version | | |

Supplementary Table 3 PlaVac UK semi-structured interview topic guide

| Topic | Guiding questions | Possible further follow-up questions |
| --- | --- | --- |
| General introduction | Tell me how you heard about the study | What was good about the (method of advertising that recruited e.g. “facebook ad” )  Was there anything in the (method of advertising that recruited) that made you particularly interested in this study? |
| Motivations | What made you want to participate in the PlaVac trial? | If you had to pick your number one reason for volunteering, could you do that? If yes/no, can you tell me more about that? |
|  | Why do you think most people volunteer for clinical trials? | Tell me more about that |
|  | Did the COVID-19 pandemic have an effect on your decision to volunteer? | Did the COVID-19 pandemic raise your awareness of clinical trials in general?  Do you think that the COVID-19 pandemic has raised awareness of clinical trials in general? |
|  | Was there anything that concerned you, or that you considered, about being in a clinical trial during a pandemic? |  |
|  | Do you think that the study reimbursement influenced your decision to take part? | Would you have taken part in the study if there was no reimbursement?  Do you think that the reimbursement amounts are fair/too high/too low? Why? |
|  | Do you think that the study being run by Oxford University had an effect on your decision to take part? | Would you “trust” any organisation running a clinical trial or are there organisations that you would trust more or less? |
|  | Did this study being for a vaccine against “the plague” impact your decision to volunteer? | What did you know about plague before you joined the trial?  Has that changed? |
| Prior experience/knowledge | Had you ever been in any other studies or clinical trials? | Tell me more about that experience  How does that experience compare to your experience of this study? |
|  | How much did you know about clinical trials before you joined this one? | Was there anything that surprised you or was different to what you expected? |
| Risk | When you decided to take part in the trial, how risky did you think it was? | What is an acceptable level of risk for taking part in a vaccine trial? |
|  | Did you discuss whether you should take part in the trial with anyone else? | How did they respond? |
|  | Did your opinion of the risks of the trial change after the screening visit at all? | Was there anything discussed in the screening visit informed consent that surprised you or made you concerned? |
|  | Were there any particular potential side effects that you worried about? | Did you ever worry that you might become seriously unwell as a result of being in the study? |
|  | How did you feel about the plague study vaccine being based on the same technology as the Oxford/AstraZeneca COVID-19 vaccine? | Was there anything that worried/reassured you about that? |
|  | What do you think your general attitude to risk is in life? | Would you describe yourself as a risk-taker?  Do you think your attitude to risk relates to your decision to join the study?  Are there any kinds of research study that you would definitely not volunteer for? |
| Donation attitudes | Have you ever considered whether you would want to be an organ donor? |  |
|  | Have you ever been a blood donor? | Do you think that being/not-being a blood donor has made you more/less likely to join a study like this? |
| Media perception and experience | How do you think that clinical trials are represented in the media? | Do you think that has changed since the pandemic? |
|  | Were you aware of this trial being in the media? | Do you think that it was presented accurately? |
| Study experience | How would you summarise your experience of the study so far? | What has been the worst aspect of taking part?  What has been the best aspect of taking part? |
|  | How has your experience of study aspects involving needles been? (blood taking and vaccination) | Did you think you would find having blood taken/vaccinations better or worse than it has been? |
|  | Does your experience of the study match your expectations of how it would be? | Is there anything that was worse/better than you thought it would be?  Is there anything that surprised you about being in the study? |
|  | What would you tell someone else about taking part in this kind of study? | Is there anything about the study that you would change if you could? |
|  | Do you think you will do any more studies in future? | (If yes) Have you thought about what kind of studies?  Can you tell me more about your reasons for wanting to do more studies?  (If no) Can you tell me more about your reasons for not wanting to do any more studies? |

Supplementary Table 4 PlaVac Uganda semi-structured interview topic guide. Questions that have been added or modified from UK version are shown in italics.

| Topic | Guiding questions | Possible further follow-up questions |
| --- | --- | --- |
| Social Demographic Characteristics | Please tell me a little bit about yourself | When you were born.  Education level, Marital Status, religion and occupation. |
| General introduction | Tell me how you heard about the study | What was good about the (method of advertising that recruited *e.g. community engagement*)  Was there anything in the (method of advertising that recruited) that made you particularly interested in this study? |
| Motivations | What made you want to participate in the PlaVac Uganda trial? | If you had to pick your number one reason for volunteering, could you do that? If yes/no, can you tell me more about that? |
|  | Why do you think most people volunteer for clinical trials? | Tell me more about that |
|  | Did the COVID-19 pandemic have an effect on your decision to volunteer? | Did the COVID-19 pandemic raise your awareness of clinical trials in general?  Do you think that the COVID-19 pandemic has raised awareness of clinical trials in general? |
|  | Was there anything that concerned you, or that you considered, about being in a clinical trial during a pandemic? |  |
|  | Do you think that the study reimbursement (payment/compensation) affected your decision to take part? | Would you have taken part in the study if there was no reimbursement?  Do you think that the reimbursement amounts are fair/too high/too low? Why? |
|  | Do you think that the study being run by the *MRC/UVRI* had an effect on your decision to take part? | Would you “trust” any organisation running a clinical trial or are there organisations that you would trust more or less? |
| Knowledge/opinion of plague | Have you ever heard of plague as a disease? | What do you know about it?  Mode of transmission, signs and symptoms, prevention methods etc…  Do you think you can die from plague?  Do you think plague can be cured? How? |
|  | Had you ever heard about plague before this trial? | What had you heard?  Where/who did you hear about plague from? |
|  | Do you think plague is an important problem in Uganda? | Tell me more about why you think that |
|  | Did this study being for a vaccine against “the plague” impact your decision to volunteer? |  |
| Prior experience/knowledge of clinical trials | Had you ever been in any other studies or clinical trials? | Tell me more about that experience.  How does that experience compare to your experience of this study? |
|  | How much did you know about clinical trials before you joined this one? | Was there anything that surprised you or was different to what you expected? |
| Risk | When you decided to take part in the trial, how risky did you think it was? | What is an acceptable level of risk for taking part in a vaccine trial? |
|  | Did you discuss whether you should take part in the trial with anyone else? | How did they respond? |
|  | Did your opinion of the risks of the trial change after the screening visit at all? | Was there anything discussed in the screening visit informed consent that surprised you or made you concerned? |
|  | Were there any particular potential side effects that you worried about? | Did you ever worry that you might become seriously unwell as a result of being in the study? |
|  | How did you feel about the plague study vaccine being based on the same technology as the Oxford/AstraZeneca COVID-19 vaccine? | Was there anything that worried/reassured you about that? |
|  | What do you think your general attitude to risk is in life? | Would you describe yourself as a risk-taker?  Do you think your attitude to risk relates to your decision to join the study?  Are there any kinds of research study that you would definitely not volunteer for? |
| Donation attitudes |  |  |
|  | Have you ever been a blood donor? | Do you think that being/not-being a blood donor has made you more/less likely to join a study like this? |
|  | Have you heard about organ donation in Uganda? | What do you think about it?  Do you think it is something you would consider doing? |
|  | How do you feel about donating blood samples for this study? | Have you ever had any worries about donating blood samples for research studies? |
| Media perception and experience | How do you think that clinical trials are represented in the media? | Do you think that has changed since the pandemic? |
|  | Were you aware of this trial being in the media? | Do you think that it was presented accurately? |
| Study experience | How would you summarise your experience of the study so far? | What has been the worst aspect of taking part?  What has been the best aspect of taking part? |
|  | How has your experience of study aspects involving needles been? (blood taking and vaccination) | Did you think you would find having blood taken/vaccinations better or worse than it has been? |
|  | Does your experience of the study match your expectations of how it would be? | Is there anything that was worse/better than you thought it would be?  Is there anything that surprised you about being in the study? |
|  | What would you tell someone else about taking part in this kind of study? | Is there anything about the study that you would change if you could? |
|  | Do you think you will do any more studies in future? | (If yes) Have you thought about what kind of studies?  Can you tell me more about your reasons for wanting to do more studies?  (If no) Can you tell me more about your reasons for not wanting to do any more studies? |
| Challenges and recommendations | What are some of the challenges that you faced while participating in this trial? | Probes: Delays in attending to him/her, fear of the injection, long distance, rumours etc… |
|  | How were you able to negotiate through these challenges? | Probe: counselling from the study staff, more information from friends, Health Education etc.. |
|  | Would you recommend your friend to participate in a vaccine trial? | What would you tell him or her? |
|  | Is there anything else that you would like to tell about the PlaVac Study? | Anything you had forgotten or important to say about the study…. |

Supplementary Table 5 Demographics of PlaVac UK and Uganda participants, entire cohorts.

|  | PlaVac UK | PlaVac Uganda |
| --- | --- | --- |
| N enrolled | 45 | 36 |
| Sex, female | 15 (33.3%) | 10 (27.8%) |
| Age at enrolment in years, median (IQR) | 38.0 (26.0-48.0) | 28.2 (24.4-34.0) |
| Ethnicicity |  |  |
| White | 42 (93.3%) | 0 (0.0%) |
| Black | 0 (0.0%) | 36 (100.0%) |
| Asian | 0 (0.0%) | 0 (0.0%) |
| Mixed | 3 (6.7%) | 0 (0.0%) |
| Other | 0 (0.0%) | 0 (0.0%) |

Supplementary Table 6 COVID-19 vaccination status of participants in PlaVac UK and Uganda trials at time of enrolment. Denotes received ≥1 dose of COVID-19 vaccine

| Vaccine received | PlaVac UK | PlaVac Uganda |
| --- | --- | --- |
| mRNA COVID-19 vaccine | 23 (51.1%) | 3 (8%) |
| ChAdOx1 nCoV-19 | 22 (48.9%) | 8 (22.2%) |
| Unvaccinated | 0 (0.0%) | 25 (69.4%) |
| Total of cohort vaccinated | 45 (100.0%) | 11 (30.6%) |
